# Supplementary material for: Comprehensive Clinical Genetics, Molecular and Pathological Evaluation Efficiently Assist Diagnostics and Therapy Selection in Breast Cancer Patients with Hereditary Genetic Background
Source: Int J Mol Sci. 2024 Nov 22;25(23):12546. doi: 10.3390/ijms252312546 (PMC11641531; doi:10.3390/ijms252312546)
Supplement: Supplementary file 1 [file ijms-25-12546-s001.zip › Suppl. Methods& Tables.pdf]

Supplementary methods

Splice effect testing

For splice effect testing RNA was isolated from blood samples collected in Tempus Blood RNA tubes (#4342792, Thermo Fisher Scientific, Waltham, MA, USA). RNA extraction was performed using Tempus™ Spin RNA Isolation Kit (#4380204, Thermo Fisher Scientific, Waltham, MA, USA) according to the manufacturer's instruction. Complementary DNA (cDNA) was synthesized via reverse transcription using ProtoScript® II Reverse Transcriptase (#E6560S, New England Biolabs, Ipswich, MA, USA) and random hexamer primers (please find primer sequences in the Supplementray Methods). For PCR amplification primers were designed using Primer3 Plus. Sequence of primers used: PMS2-ex6-F: AGGAAAATATCGGCTCTGTGTTT, PMS2-ex10-R: TGCCTTTTATCTGGAGTAACATTGAT. PCR products were assessed on 1.5% agarose gel electrophoresis against control samples using HyperLadder™ 1kb (#BIO-33053, Meridian Bioscience, Cincinnati, OH, USA) for reference. Purified PCR products were analysed by Sanger sequencing.

NGS data analysis

Nucleotides with a Phred quality score of a minimum of 30 per position were accepted. Reads were aligned to the GRCh37 human genome assembly. Coding regions (exons) ± 30 bp were analysed.

In mapping metrics, the input read number was an average 2.5 million/sample, and the average ratio of proper reads was 95%. Heterozygous loci were accepted with a variant allele frequency (VAF) between 30 and 70%. The average base coverage for HBOC genes was 208 with a minimum of 77 and maximum of 594 reads/base. Low-covered bases (<10 reads/bp) accounted for an average of 0.1% per gene. For CNVs, we applied Illumina BaseSpace Dragen Enrichment v.4.03-v.4.2.7 algorithm as caller. We used a sensitive setting where copy ratio – CR – filter threshold 0.2 led to calls less than CR=0.8 or greater than CR=1.2. By applying this filter (high sensitivity, low specificity) we could avoid false negative cases.

MLPA probes used for validation

(P043-E1 APC; P041-B1 ATM-1; P042-B2 ATM-2; P002-D1 BRCA1; P045-D1 BRCA2/CHEK2; P083-D2 CDH1; P190-D1; CHEK2; P003-D1 MLH1-MSH2; P072-D1 MSH6-MUTYH; P081-D1 NF1 mix 1; P082-C2 NF1 mix 2; P260-C1 PALB2-RAD50-RAD51C-RAD51D; P008-C1 PMS2; P158-D1 JPS; P226-D1 SDH; P056-D1 TP53; P046-D1 TSC2, MRC-Holland, Amsterdam, The Netherlands).

Variant interpretation

The clinical significance of the variants was interpreted based on The American College of Medical Genetics and Genomics (ACMG) guidelines including patient phenotype and family history [50–52] using Varsome (https://varsome.com/), and Franklin (https://franklin.genoox.com/clinical-db/home) platforms. For gene-specific classification the National Institutes of Health (NIH)-funded ClinGen Evidence Repository (an FDA-recognized human genetic variant database containing expert-curated assertions regarding variants' pathogenicity and supporting evidence summaries (https://clinicalgenome.org/), along with BRCA Exchange (https://brcaexchange.org/) and NCBI ClinVar (https://www.ncbi.nlm.nih.gov/clinvar/) databases and extensive literature search were used.

Regarding CHEK2 gene, following NCCN guidelines [Daly et al.] and recent data [Bychkovsky et al.] three low penetrance missense CHEK2 variants ((NM_007194.4):c.470T>C, p.Ile157Thr; c.1283C>T, p.Ser428Phe; c.1427C>T p.Thr476Met) do not pose a risk for breast cancer and thus do not affect patient management. Therefore, these variants were excluded from our analysis.

Daly, M.B.; Pal, T.; Berry, M.P.; Buys, S.S.; Dickson, P.; Domchek, S.M.; Elkhanany, A.; Friedman, S.; Goggins, M.; Hutton, M.L.; et al. Genetic/Familial High-Risk Assessment: Breast, Ovarian, and Pancreatic, Version 2.2021, NCCN Clinical Practice Guidelines in Oncology. J. Natl. Compr. Cancer Netw. JNCCN 2021, 19, 77–102, doi:10.6004/jnccn.2021.0001

Bychkovsky, B.L.; Agaoglu, N.B.; Horton, C.; Zhou, J.; Yussuf, A.; Hemyari, P.; Richardson, M.E.; Young, C.; LaDuca, H.; McGuinness, D.L.; et al. Differences in Cancer Phenotypes Among Frequent CHEK2 Variants and Implications for Clinical Care-Checking CHEK2. JAMA Oncol. 2022, 8, 1598–1606, doi:10.1001/jamaoncol.2022.4071.

Supplementary Table 1. Breast cancer patients with mismatch repair gene deficiency. All patients met the testing criteria of HBOC genetic testing. Out of the 7 cases, 5 breast cancer tissue specimens were available. In 4 tumours MMR deficiencies were observed by immunhistochemistry, while in 1 case MSH2 staining was present.

MMR: mismatch repair; n.a: not available; HBOC: hereditary breast and ovarian cancer

**Germline heterozygote P/LP MMR gene variants**	**MMR protein Immunohistochemistry on breast cancer tissue**	**Proband phenotype**	**Family history (among 1-3 degree relatives)**	**Lynch syndrome could be suspected baed on personal and family history**	**Association of breast cancer with MMR gene defect**
MSH6(NM_000179.3):<br>c.3261dup<br>p.(Phe1088Leufs*5)	isolated MSH6 loss	ovarian cancer (49 y)	ovarian cancer (48 y),<br>colorectal cancer (60 y)	no	yes
PMS2(NM_000535.7):<br>c.903+3A>G<br>p.(?)	isolated PMS2 loss	breast cancer (47 y)	3 cases of breast cancer (78 y, 57 y, 81 y)	no	yes
MSH2(NM_000251.3):<br>c.586C>T<br>p.(Pro196Ser)	no specimen available	breast cancer (38 y)	breast cancer (64 y)	no	n.a
MSH2(NM_000251.3):<br>c.873_876del<br>p.(Thr292Leufs*8)	MSH2/MSH6 loss	colorectal cancer (35 y),<br>edometrial cancer (43 y),<br>breast cancer (TNBC) (49 y)	endometrial cancer (38 y)<br>6 cases of colorectal cancer (age: n.a.),<br>2 cases of gastric cancer (age: n.a.),<br>breast cancer (age: n.a.)	yes	yes
MSH2(NM_000251.3):<br>c.1226_1227del<br>p.(Gln409Argfs*7)	MSH2/MSH6 loss	breast cancer (57 y),<br>endometrial cancer (65 y),<br>colorectal cancer (65 y)	colorectal cancer (39 y)	yes	yes
MLH1(NM_000249.4):<br>c.870dupA<br>p.(Phe291Ilefs*16)	no specimen available	endometrial cancer (50 y),<br>colorectal cancer (71 y),<br>breast cancer (78 y)	2 colorectal cancer cases (42 y, 51 y),<br>3 endometrial cancer cases (35 y, 44 y, 44 y)<br>1 ovarian cancer at age 56	yes	n.a.
MSH2(NM_000251.3):<br>c.586C>T<br>p.(Pro196Ser)	positive MSH2 staining, no MSH2 loss was detected	breast cancer (38 y)	breast cancer (64 y)	no	no

Supplementary Table 2. Phenotype and genetic variants identified in patients not meeting NCCN genetic testing criteria

Proband's genotype	Proband's phenotype	Cancer cases in family
ATM (NM_000051.4):<br>c.7096G>T<br>p.(Glu2366Ter)	Breast cancer at age 65	1 breast cancer >60 years
CHEK2(NM_007194.4):<br>del(ex 9-11)<br>p.(?)	Breast cancers at age 62 and 75	No affected family members
MSH2(NM_000251.3):<br>c.1226_1227del<br>p.(Gln409fs)	Breast cancer at age 57,<br>Endometrial cancer at age 65,<br>Gastric cancer at age 78	1 colorectal cancer

Supplementary Table 3. Clinicopathological parameters of first tumours in probands according to genotype

	**Probands who did not meet NCCN criteria for HBOC**	**Probands with P/LP variants in any HBOC gene**	**Probands with no P/LP variants**	**Probands with P/LP variants in any high penetrance gene**	**Probands with P/LP *BRCA1* variants**	**Probands with P/LP *BRCA2* variants**	**Probands with P/LP *PALB2* variants**	**P/LP variants in other high penetrance genes**	**Probands with P/LP variants in any moderate penetrance gene**	**Probands with P/LP *ATM* variants**	**Probands with P/LP *CHEK2* variants**
**Clinicopathological parameter**											
**Number of probands (n)**	50	96	367	67	32	24	7	4	29	6	10
**Age at cancer onset (years)**											
**Breast cancer-female (mean ±SD)**	56.57±8.30	44.41±9.96	48.12±11.38	43.55±10.48	41.11±9.89	47.38±10.89	41.67±9.95	43.33±11.37	46.26±8.61	47.20±10.01	41.60±3.84
**MBC (mean±SD)**	0	57.00	68.00±10.72	57.00	0	0	57	0	0	0	0
**Ovarian cancer (mean±SD)**	0	53.57±10.23	53.39±13.93	50.00±8.29	49.33±9.45	51.00	0	0	62.50	68.00	0
**Pancreatic cancer (mean±SD)**	0	52.00	62.38±10.38	52.00	0	50.00	54.00	0	0	0	0
**Prostate cancer (mean±SD)**	65.00±6.75	64.00	63.50±4.99	64.00	0	0	0	64.00	0	0	0
**Ki 67 of breast cancer (mean±SD)**	26.07±21.04	41.18±26.46	30.28±25.51	45.02±26.95	59.11±26.01	37.19±23.01	21.5±10.10	20.00±8.66	31.23±22.81	31.25±14.36	30.00±19.36
**ER pos proportion (95%CI)**	4 (0.66-0.89)	**1.15 (0.43-0.64)***	2.25 (0.64-0.74)	**0.75 (0.59-0.86)***	**0.15 (0.06-0.34)***	2.14 (0.47-0.84)	5.00 (0.42-0.99)	1.50 (0.23-0.89)	4.600 (0.64-0.93)	4.00 (0.36-0.98)	7.00(0.60-1.00)
**PR pos proportion (95%CI)**	1.81 (0.50-0.77)	1.02 (0.40-0.61)	1.57 (0.56-0.66)	**0.63 (0.47-0.77)***	**0.12 (0.03-0.30)***	1.75 (0.43-0.80)	5.00 (0.42-0.99)	0.67 (0.20-0.94)	3.67 (0.60-0.90)	4.00 (0.36-0.98)	7.00(0.60-1.00)
**HER2 pos proportion (95%CI)**	0.45 (0.29-0.62)	0.17 (0.10-0.28)	0.29 (0.24-0.34)	0.15 (0.08-0.27)	0.21 (0.09-0.41)	0.05 (0.00-0.25)	0.20 (0.02-0.64)	0.25 (0.03-0.71)	0.27 (0.13-0.48)	0.67 (0.20-0.94)	0.17 (0.01-0.58)
**TNBC proportion (95%CI)**	**0.00 (0-0.09)***	**0.61 (0.47-0.73)***	0.27 (0.22-0.32)	**0.91 (0.75-0.98)***	**2.50 (0.53-0.85)***	0.47 (0.25-0.70)	0.20 (0.02-0.64)	0.25 (0.03-0.71)	0.12 (0.03-0.31)	0.00 (0.00-0.49)	0.00 (0.00-0.40)
**Mpx/bilat HBOC proportion (95%CI)**	0.16 (0.08-0.30)	**0.32 (0.23-0.44)***	0.16 (0.13-0.20)	**0.35 (0.23-0.48)***	**0.52 (0.32-0.72)***	0.25 (0.11-0.47)	0.00 (0.00-0.40)	0.50 (0.15-0.85)	0.25 (0.12-0.45)	0.20 (0.02-0.64)	0.14 (0.01-0.53)
**Mpx non HBOC proportion (95%CI)**	0.13 (0.06-0.27)	0.06 (0.02-0.13)	0.06 (0.04-0.09)	0.04 (0.01-0.13)	0.03 (0.00-0.18)	0.00 (0.00-0.16)	0.17 (0.01-0.58)	0.20 (0.02-0.64)	0.11 (0.03-0.29)	0.20 (0.02-0.64)	0.00 (0.00-0.37)

*p<0,05
