# Supplementary figures and images for: Comprehensive Clinical Genetics, Molecular and Pathological Evaluation Efficiently Assist Diagnostics and Therapy Selection in Breast Cancer Patients with Hereditary Genetic Background
Source: Int J Mol Sci. 2024 Nov 22;25(23):12546. doi: 10.3390/ijms252312546 (PMC11641531; doi:10.3390/ijms252312546)

Supplementary Figure 1

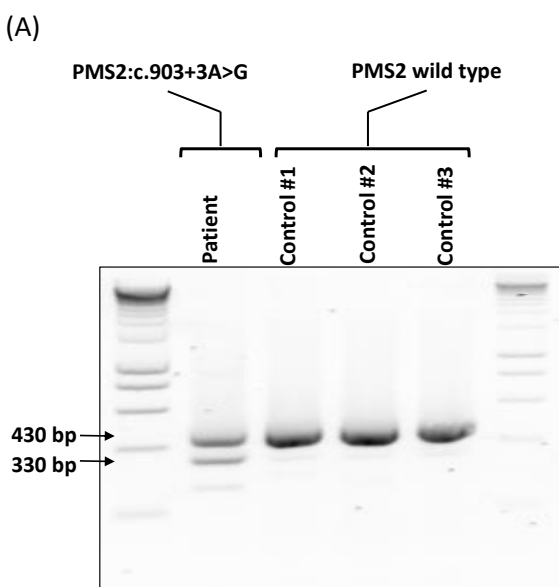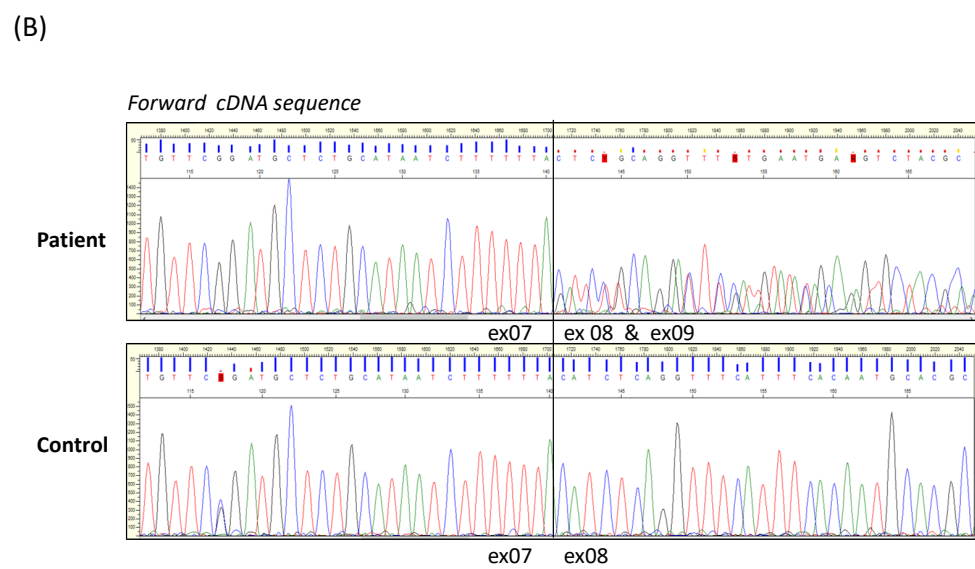

(A)

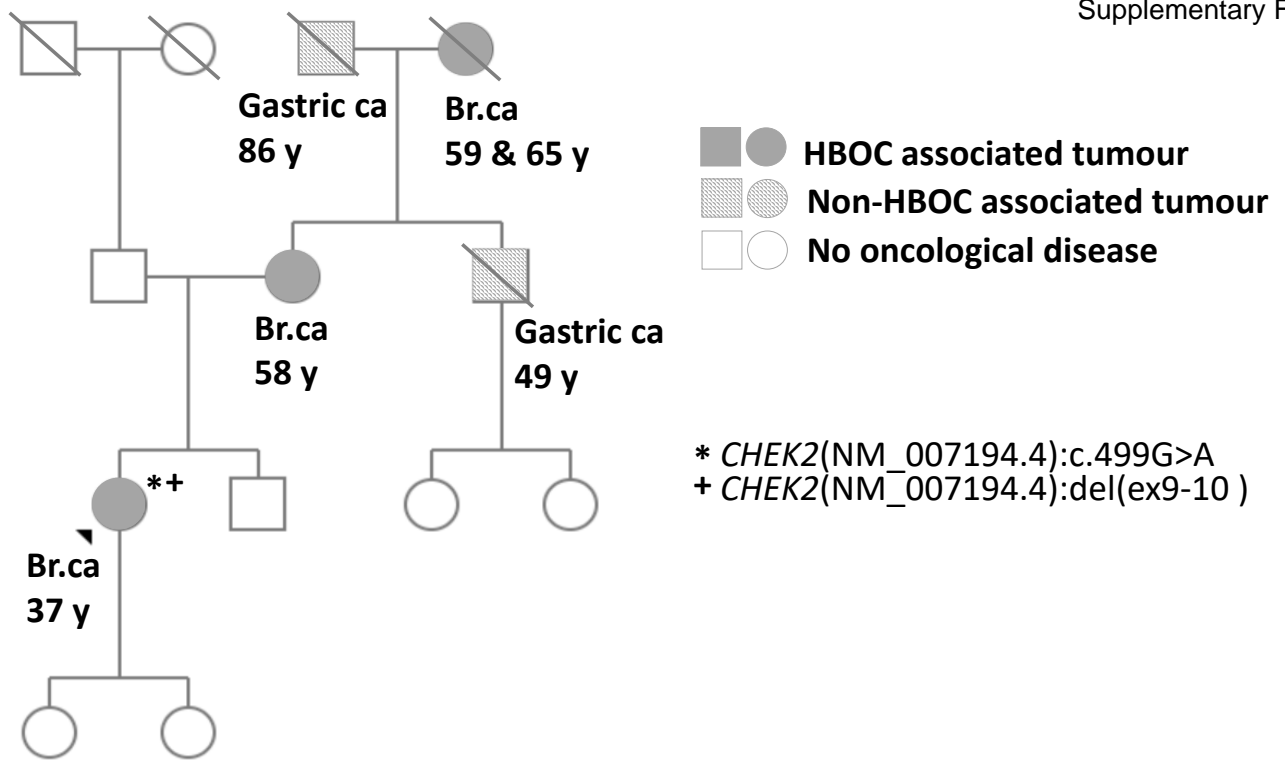

(B)

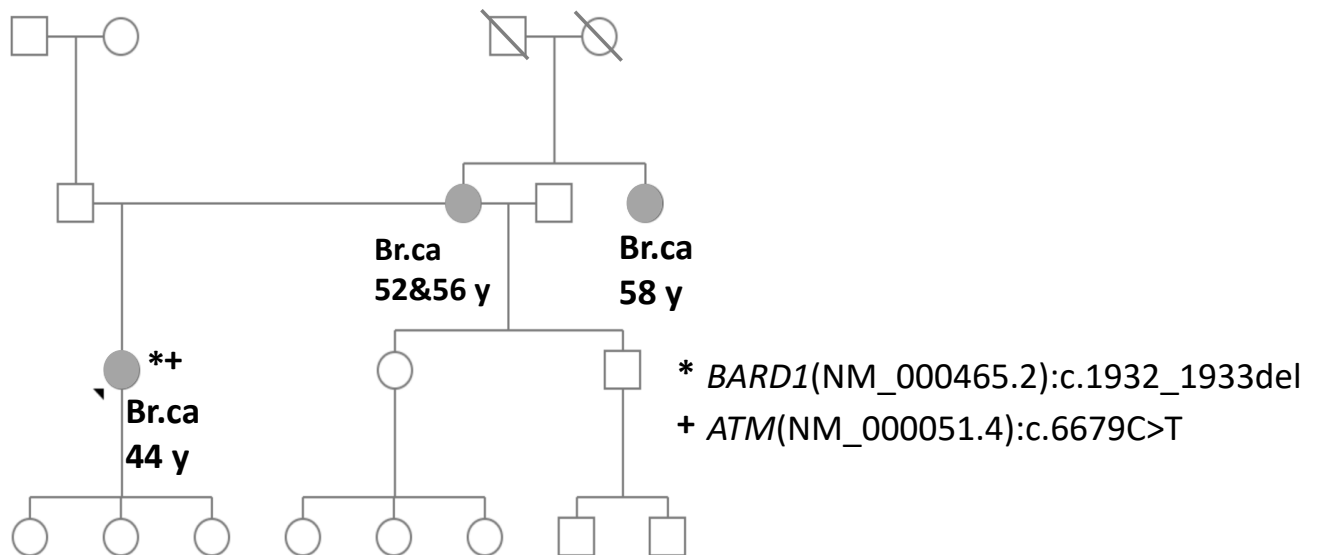

(C)

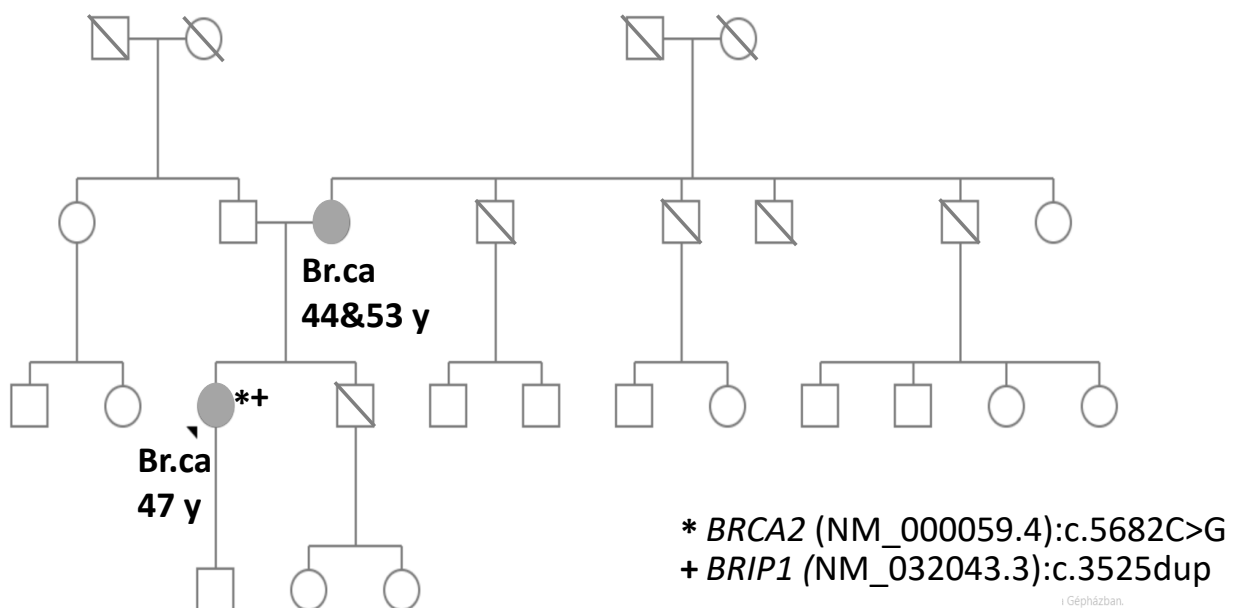

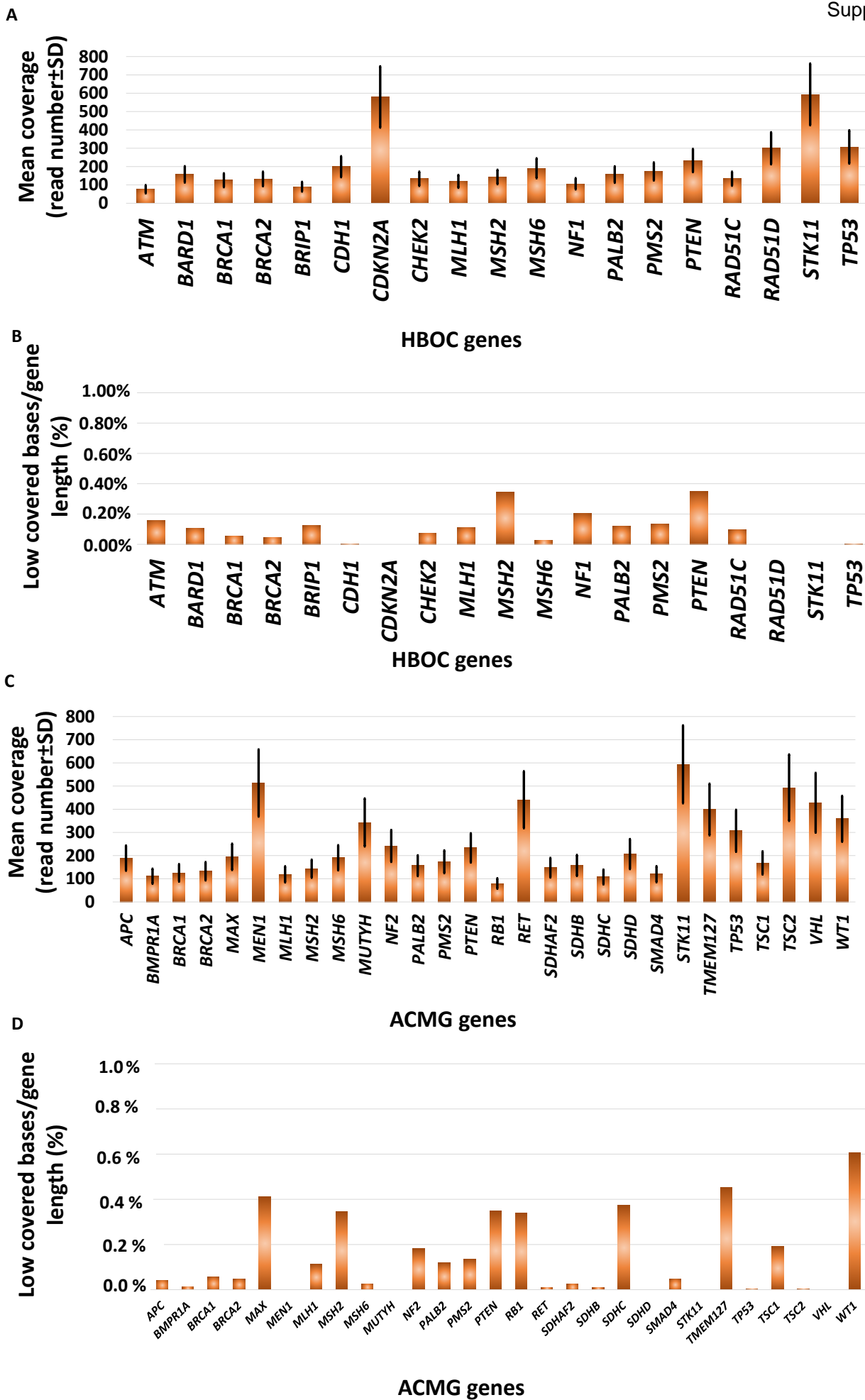

Supplement: Supplementary file 1 [file ijms-25-12546-s001.zip › merged_SFigures_leg.pdf]
